# A Comparative Study of the Impact of the Bleaching Method on the Production and Characterization of Cotton-Origin Nanocrystalline Cellulose by Acid and Enzymatic Hydrolysis

**DOI:** 10.3390/polym15163446

**Published:** 2023-08-18

**Authors:** Faik Bolat, Jana Ghitman, Madalina Ioana Necolau, Eugeniu Vasile, Horia Iovu

**Affiliations:** 1 Advanced Polymer Materials Group, National University of Science and Technology Politehnica Bucharest, 1–7 Gh. Polizu Street, 011061 Bucharest, Romania; faik.bolat@stud.chimie.upb.ro (F.B.); jana.ghitman@upb.ro (J.G.); madalina.necolau@upb.ro (M.I.N.); 2 Department of Oxide Materials Science and Engineering, National University of Science and Technology Politehnica Bucharest, 1–7 Gh. Polizu, 060042 Bucharest, Romania; eugeniuvasile@yahoo.com; 3 Academy of Romanian Scientists, 54 Splaiul Independentei, 050094 Bucharest, Romania

**Keywords:** bleaching, acid hydrolysis, enzymatic hydrolysis, cellulose nanocrystals

## Abstract

Due to environmental concerns, as well as its exceptional physical and mechanical capabilities, biodegradability, and optical and barrier qualities, nanocellulose has drawn a lot of interest as a source of reinforcing materials that are nanometer sized. This article focuses on how to manufacture cellulose nanomaterials from cotton by using different types of acids such as H_2_SO_4_ and HCI in different concentrations and in the presence of enzymes such as cellulase and xylanase. Two different types of bleaching methods were used before acid and enzyme hydrolysis. In the first method, cellulose was extracted by bleaching the cotton with H_2_O_2_. In the second method, NaOCl was utilized. For both methods, different concentrations of acids and enzymes were used to isolate nanocellulose materials, cellulose nanocrystals (CNC), and cellulose nanofibrils (CNF) at different temperatures. All obtained nanocellulose materials were analyzed through different techniques such as FT-IR, Zeta potentials, DLS, Raman spectroscopy, TGA, DSC, XRD, and SEM. The characteristic signals related to cellulose nanocrystals (CNC) were confirmed with the aid of Raman and FT-IR spectroscopy. According to the XRD results, the samples’ crystallinity percentages range from 54.1% to 63.2%. The SEM image showed that long fibers break down into small fibers and needle-like features are seen on the surface of the fibers. Using different types of bleaching has no significant effect on the thermal stability of samples. The results demonstrate a successful method for synthesizing cellulose nanofibrils (CNF) from cotton through enzymatic hydrolysis, but the results also demonstrated that the choice of bleaching method has a significant impact on the hydrodynamic properties and crystallinity of both CNC and CNF samples.

## 1. Introduction

Nanocellulose is a derivative of cellulose, one of the most abundant biopolymers, which is characterized by having at least one size on a nanoscale. The properties of nanocellulose provide new opportunities for materials science and applications. Nanocrystalline cellulose is considered one of the most important nanoparticles because it has many important practical applications, such as tremendous mechanical strength and biodegradability. It comes from a renewable resource, is inexpensive, and is environmentally friendly [1]. Due to its lightness and transparency, it has various applications, such as pharmaceuticals, food, electronics, and medicine. Any natural cellulosic source material, including wood pulp, cotton, banana peels, rice husks, and so on, can be used to produce nanocellulose through different pathways [2]. Superior properties such as high surface areas (~250 m^2^/g), high modulus (~145 GPa), and strength (~7500 MPa) make it one of the strongest and stiffest organic macromolecules [3].

Cellulose is the primary ingredient in a variety of plant fibers and waste from crops such as cotton, bananas, rice, and wheat and can exist in three main forms: cellulose, hemicellulose, and lignin [4]. The linear D-glucose units in cellulose create microfibrils with a firm, organized structure that gives the polymer its strength [5].

Hemicelluloses represent a class of polysaccharides whose structure is determined by the plant and the tissue of the plant. The most prevalent hemicellulose with a D-glucose backbone is a kind of cellulose called xyloglucan. An amorphous complex phenolic polymer called lignin is made up of phenylpropane units such as p-coumaryl, coniferyl, and sinapyl alcohol, which are cross-linked and connected in several ways [6]. The biochemical composition of plant tissue affects how difficult it is to separate lignocellulosic biomass into its constituent parts [7]. Thus, in order to improve the accessibility of cellulose and hemicelluloses in the biorefinery process, lignocellulosic biomass must be processed [8].

Textiles have been bleached for a very long time. Chlorine-containing bleaches sometimes involve adding chlorine atoms (rather than oxygen atoms) to the chemical compounds during the oxidation process. In some situations, adding chlorine results in the creation of dangerous byproducts like dioxins. Although hydrogen peroxide oxidizes by either removing hydrogen atoms or adding oxygen, it does not contain any chlorine atoms. As a result, the compound cannot include pollutants of the kind that are organochlorine, and the issue of hazardous pollution is, therefore, prevented at the source [9].

According to the literature, the pretreatment used before chemical treatments such as NaClO_2_, NaOCl, or H_2_O_2_ boosted the production of cellulose nanocrystals and enhanced their physical and mechanical qualities [10,11,12,13]. Several studies have been conducted to produce cotton cellulose nanocrystals using acid hydrolysis [1,14,15,16,17,18]. But, there is little research on using enzymatic hydrolysis to extract CNC or CNF [19,20]. In general, cellulose nanofibrils are produced by enzymatic hydrolysis processes, whereas CNCs are produced by nonenzymatic methods [21].

The main objective of this study is the extraction of nanocellulose materials (cellulose nanocrystals or nanofibrils) from commercial cotton using two distinct bleaching processes with alkali (4 wt.% NaOH) and bleaching agents (H_2_O_2_ and NaOCl), followed by acid (H_2_SO_4_ and HCl) treatment and enzymatic hydrolysis. The novel aspect of this research is comparing the traditionally used NaOCl bleaching method with the more environmentally friendly H_2_O_2_ pretreatment method to understand how different types of pretreatments and bleaching methods affect the physical and mechanical properties of extracted CNC or CNF with potential commercial applications.

## 2. Materials and Methods

### 2.1. Materials

Cotton is supplied from a local market. Sulfuric acid with a 96 wt.% concentration was supplied from SIRAL Trading SRL Romania, Toluene (99.9%). Ethyl alcohol, sodium hypochlorite (NaOCl), and sodium hydroxide were received from SC Chimreactive SRL. Hydrogen peroxide was received from S.C. SIRAL Trading SRL. Hydrochloric acid was supplied by Crystal Chim SRL. The enzyme cellulase (enzyme activity: 500,000 U/g) and xylanase (enzyme activity: 290,000 U/g) were supplied by Bengbu Milliflux Biopharma Co., Ltd. (Bengbu, China).

### 2.2. Methods

#### 2.2.1. Pretreatment of Cotton Fibers

The cotton samples from commercial purchases were cleaned with distilled water (1 g cotton:10 g water) and dried at 50 °C for 24 h. To remove lignin, cotton samples were treated with a 2:1 (*v*:*v*) toluene and ethanol mixture for 24 h [22]. The solvent and other impurities were then removed by washing the samples with a 95 wt.% C_2_H_5_OH solution, followed by 3 h of heating at 80 °C. Two different bleaching methods were then used for cotton fibers:

1st method: 20 wt.% (w) H_2_O_2_ and 4 wt.% (w) NaOH solutions were used to bleach 20 g cotton samples at 80 °C for 4 h.
2nd method:16 wt.% (w) NaOCl and 4 wt.% (w) NaOH solutions were used to bleach 20 g cotton samples at 80 °C for 4 h.

#### 2.2.2. Preparations of Nanocellulose Particles

For each bleaching method, two acid hydrolyses (H_2_SO_4_ and HCl) and one enzymatic hydrolysis method were applied to obtain nanocellulose particles (CNC or CNF).

H_2_SO_4_ hydrolysis method: 2 g of pretreated cotton samples were mixed (with a cotton-to-acid ratio is 1:50 g/mL) with 60 wt.%, 50 wt.%, and 40 wt.% H_2_SO_4_ solution (100 g) and stirred for 10, 20, and 30 min at 70 °C. To stop the hydrolysis, the suspension was then poured into 1 L of deionized water. As shown in Table 1, six distinct samples were collected [23]. The color of the obtained CNC solutions at 60 wt.% was brown.

HCl Acid Hydrolysis method: Pretreated cotton samples (2.00 g) were mixed with 10 wt.%, 15 wt.%, and 20 wt.% HCl solutions (50 mL) and stirred for 60, 40, and 20 min at 75 °C [24]. Then, the same protocol was followed as in the H_2_SO_4_ acid hydrolysis method, and six different samples were obtained (Table 2).

Less hydrolysis time was used for high acid concentrations in both acid hydrolysis reactions. Depending on the pH, an alkaline (5% ammonia) or acid solution (10% acetic acid) was used to neutralize the insoluble residue after each acid hydrolysis stage, which was then thoroughly cleaned with deionized water until neutral pH and centrifuged (9000 rpm for 5 min). When aqueous solutions of acetic acid and ammonia are mixed, the resulting neutralization reaction produces highly ionized salt of ammonium acetate which can be filtered easily. Before further analysis, the collected CNC was washed, centrifuged five times, and then freeze-dried. The freeze-dried CNC exhibited a flake-like structure with some micron-sized fibrillar particles.

Enzymatic Hydrolysis Method: The experiments were conducted in 500 mL Erlenmeyer flasks containing 1 g of pretreated cotton fibers and 100 mL of sodium citrate buffer at pH 4.0. At a speed of 200 rpm, the suspension was blended at 50 °C for 30 min [25]. Over the process, various volumes of the enzymatic mixture (xylanases and cellulases) were added to the suspension at 50 °C and pH 4.0. (24 h). To end the reaction, the medium was heated at 96 °C for 15 min and the six collected samples are summarized in Table 3.

**Figure 1:**
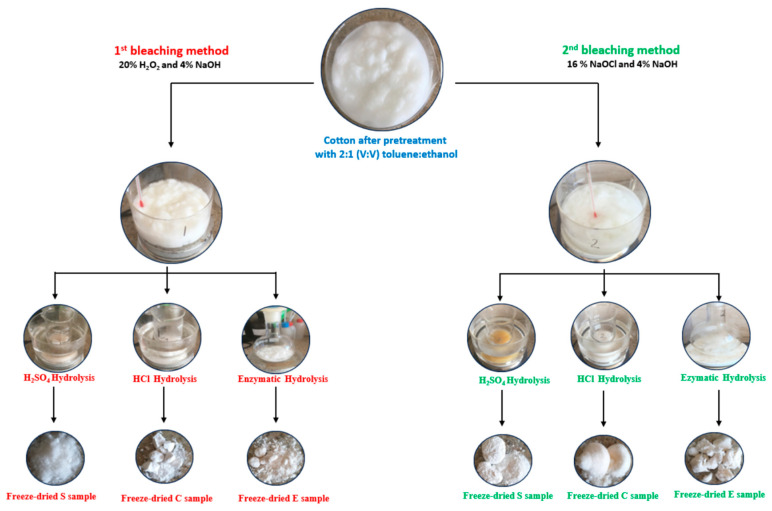
Nanocellulose extraction process.

The extraction process of nanocellulose through the two methods is schematically summarized in Figure 1.

## 3. Characterization Techniques

### 3.1. Zeta Potentials (ZP)

Using a Malvern Zetasizer Nano particle analyzer and assuming Smoluchowski behavior, the surface potential and colloidal stability of CNC particles are assessed in zeta potential [26]. Due to the electrostatic attraction between the particles, nanoparticle suspensions become more stable. The charge stability of nanoparticles in a suspension is determined by the zeta potential value. The greater the absolute value of zeta potential for nanoparticles with a negative surface charge, such as sulfate ions, indicates a better level of stability for the particles [26]. Thus, 10 mg of the obtained 18 samples (6 HCl, 6 H_2_SO_4_ acid hydrolysis, and 6 enzymatic hydrolysis samples) were suspended in 10 mL of filtered water to prepare 0.1% wt. CNC suspensions. Each sample was sonicated for 20 min at 40% amplitude and analyzed for zeta potential (ZP). The experiments were carried out at a temperature of 25 °C, and the zeta potentials that were reported are the mean findings of three measurements with 25 consecutive cycles. Among 18 samples, the best promising 6 samples were chosen for further analyses (1S_1_, 2S_1_, 1C_3_, 2C_3_, 1E_3_, and 2E_3_)

### 3.2. Dynamic Light Scattering (DLS) Measurements

DLS is an effective tool for measuring the mean hydrodynamic diameter (d, nm) and distribution of particles (PdI). An incident laser beam can be scattered by nanoparticles, which are dispersed in the colloidal system, and the scattered light is detected in the DLS system [27]. Using a Malvern Zetasizer Nano particle analyzer at 25 °C, 3 tests with 15 consecutive cycles were conducted to determine hydrodynamic “apparent particle size” and PdI in 0.1 wt.% CNC dispersions.

### 3.3. FTIR Analysis

The chemical composition of the chosen samples was investigated using FTIR analysis on a Vertex 70 Bruker FTIR spectrometer with an attenuated total reflectance (ATR) accessory. A total of 32 scans were registered in the ATR-FTIR mode for each sample, with a resolution of 4 cm^−1^ in the 600–4000 cm^−1^ wavenumber range.

### 3.4. Raman Spectroscopy

The Raman spectra were obtained using a Renishaw in Via Raman microscope system (473 nm laser excitation, Renishaw), outfitted with a 100 objective at a 0.4 mW incident power. The spectra were gathered in the range of 1000–3200 cm^−1^.

### 3.5. X-ray Diffraction (XRD) Spectroscopy

X-ray diffraction analyses (XRD) were performed using a Panalytical X’PERT MPD X-ray Diffractometer (Malvern Panalytical, Royston, UK) with Cu-K radiation operating (λ = 1.5418 Å) in the range 2θ = 2–60° to determine the crystallinity of the materials.

### 3.6. Scanning Electron Microscopy (SEM)

A field emission gun (FEG) with a resolution of 1.2 nm was used for the SEM studies on a Quanta Inspect F50 (Hillsboro, OR, USA). Using a field emission gun that was operating at 30 kV accelerating voltage, the dried samples were sputter-coated with a thin coating of gold. Sample photos were then captured at various magnifications to examine the morphology of the samples.

### 3.7. TGA Analysis

A TGA Q500 V20.10 Build 36 equipment was used to perform a thermogravimetric analysis (TGA) to determine the materials’ thermal stability. A platinum crucible containing samples with a mass of around 3–5 mg was used to record thermograms from 20 °C to 600 °C at a heating rate of 10 °C/min and nitrogen flow of 10 mL/min.

### 3.8. DSC Analysis

A Netzsch DSC 204 F1 Phoenix differential scanning calorimeter was used to perform the calorimetric experiments, with nitrogen flowing at a constant rate of 20 mL/min. The DSC thermograms were recorded using a heating rate of 10 °C/min on samples weighing about 8 mg that were put in aluminum pans. The thermograms were recorded in the 20 °C to 400 °C range.

## 4. Results

### 4.1. Hydrodynamic Characteristics and Zeta Potential of the Investigated CNC Samples

The potential difference between the stationary liquid layer affixed to the dispersed particle and the dispersion medium is known as the zeta potential. It is a potential for colloidal particles in an electric field in the shear plane [28]. The high zeta potential will provide stability for very small molecules and particles, which means the solution or dispersion will be resistant to deposition. When the potential is low, the attractive forces can overcome the thrust, breaking the dispersion, and causing the particles to clump together. As a result, low zeta potential colloids tend to clot or agglomerate, as illustrated in Table 4, whereas high zeta potential (negative or positive) colloids are electrically stabilized.

**Figure 2:**
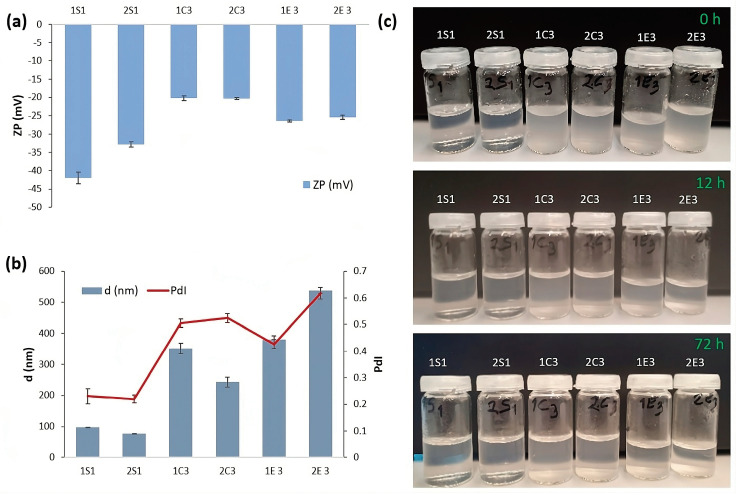
(**a**) Zeta potential for extracted CNCs in a 0.1 wt.% dispersion; (**b**) mean diameter and PdI and (**c**) photographs of apparent stability of all the investigated samples after 72 h.

The measurement of zeta potential is a straightforward method for estimating the stability of CNC suspensions, which is critical for nanocomposite materials. Figure 2a displays the average zeta potential of three different types of CNC or CNF particles (HCl hydrolysis, H_2_SO_4_ hydrolysis, and enzymatic hydrolysis), ranging from −20.2 mV to −42 mV, with the highest stability registered for 1S_1_ and 2S_1_ samples (PZ between −42 mV and −32.8 mV, respectively). It has been determined from previous studies that increasing acid concentration, duration, and temperature causes hydrolysis to occur more effectively. When the temperature of the acid is around 70 °C and more concentrated sulfuric acid is used (55% and more), the color of the product turns black [30]. In the same research, it was found that the zeta potential of CNC is −29.7 mV by using 65% sulfuric acid, during 50 min at 55 °C, but in our study, we used only a 10-min hydrolysis time at 70 °C by using a 60% sulfuric acid solution. Considering the zeta potential of CNCs extracted by the HCl hydrolysis method, these samples show fewer stable suspensions (PZ ranges between −20.2 and −20.3). The main cause of this is the lack of negative charges on the surface of CNCs produced by HCl hydrolysis. Similar results were found by Sánchez et al. [31].

On the other hand, 1E_3_ and 2E_3_ samples (−26.4 and −25.4, respectively) exhibit slightly higher stability than 1C_3_ and 2C_3_ samples. This is due to the citrate ions used as a buffer solution during the enzymatic hydrolysis of cotton. This work showed very similar results to De Aguiar et al. [25] who reported a zeta potential value of CNCs, which were obtained from sugarcane bagasse and straw, of −25 mV, showing a colloidal suspension with moderate stability, whereas in another work, Chumchoochart et al. [32] reported a high ZP value of −45.8 mV by using the enzymatic hydrolysis method.

Figure 2b demonstrates that the mean hydrodynamic diameter sizes of the 6 CNC samples range from 77 nm to 539 nm, indicating a broad diversity of lengths. The samples from H_2_SO_4_ hydrolysis (1S_1_ and 2S_1_) had the smallest particles, measuring 97.91 and 77.17 nm, respectively. (Figure 2b). The main reason for the larger particle sizes in the HCl hydrolysis samples (1C_3_ and 2C_3_), which are 350.8 and 243.1 nm, respectively, would be the following: 1S_1_ and 2S_1_ samples are prepared using 60% H_2_SO_4_ (the highest concentration among S samples), whereas 1C_3_ and 2C_3_ samples are prepared using 10% HCl (lowest concentration among C samples). In both samples, the 2nd method shows smaller particle size proving that the NaOCl bleaching method is more effective than the H_2_O_2_ bleaching method in terms of hydrodynamic diameter sizes. Similar size distributions were also observed in the work of Sánchez et al. [31] who obtained larger CNC-C sample particle sizes (300–800 nm), compared to CNC-S samples (100–500 nm). Further, 1E_3_ and 2E_3_ samples have the biggest particle sizes—380.2 nm and 538.7 nm, respectively.

Figure 2c displays the images that demonstrate the stability of all 6 samples in aqueous suspension after being sonicated for 20 min at 40% amplitude. The suspensions were photographed at 0 h, 12 h, and 72 h. At first, all samples are colorless and homogenous, and no sedimentation was observed at the bottom of the bottle; but, over a considerable period (72 h), sedimentations happen in the 1E_3_ and 2E_3_ samples (due to large mean diameter and reduced ZP values), whereas the good stability for samples 1S_1_ and 2S_1_ are maintained even after 72 h. Among the six samples, 1S_1_ and 2S_2_ samples showed the best homogeneous suspension due to the electrostatic repulsion forces between sulfate groups in CNC surfaces, in accordance with zeta potential and DLS results.

### 4.2. FTIR Analysis

**Figure 3:**
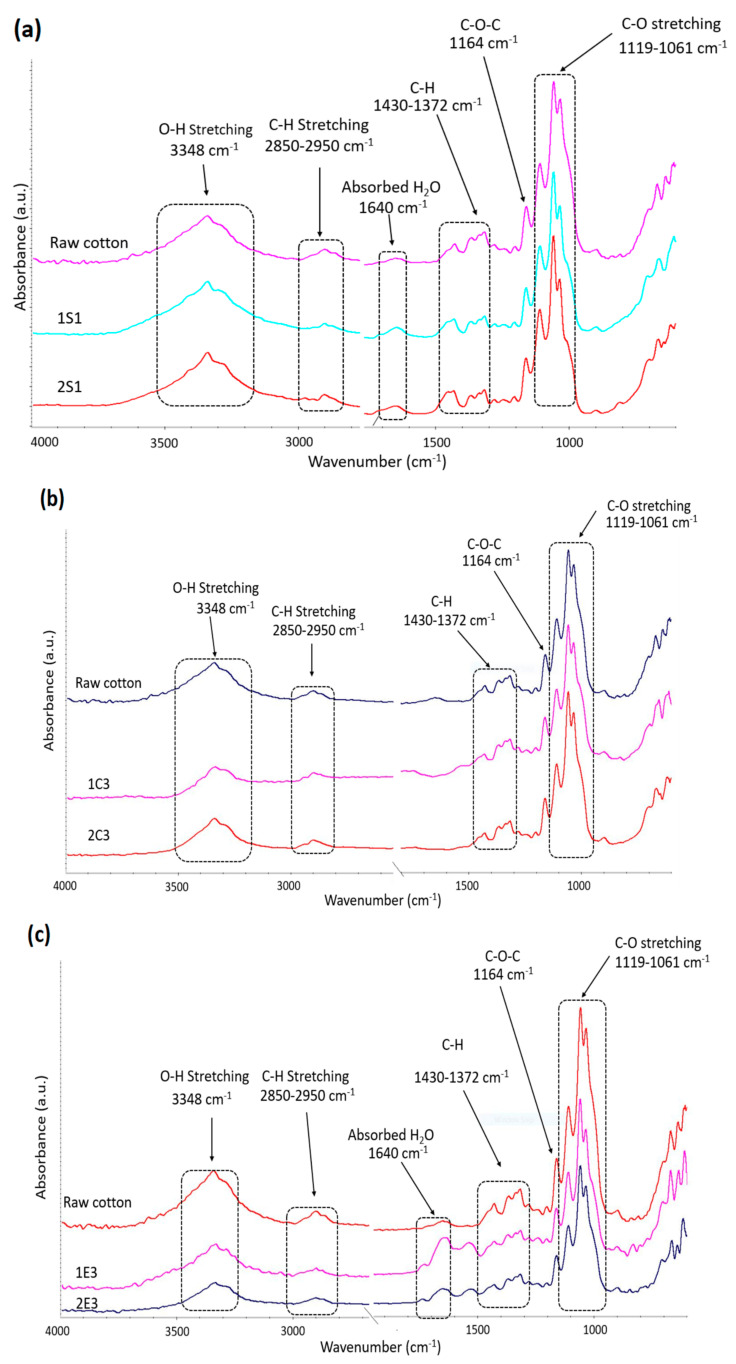
FTIR spectra of CNC samples obtained through (**a**) H_2_SO_4_ hydrolysis; (**b**) HCl and (**c**) enzymatic hydrolysis along with raw cotton.

FTIR was used to examine the chemical structure of the synthesized CNC samples and raw cotton and the corresponding spectra are presented in Figure 3.

The broad absorbance peaks at 3400–3300 cm^−1^ areas that are present in all the recorded spectra are due to the stretching and bending vibrations of the cellulose OH groups. The absorption peak at that area is known as the crystalline band. The peaks at 2900–2800 cm^−1^ correspond to CH stretching, while the peak around 1400 cm^−1^ may be assigned to CH_2_ bending vibration [33]. The asymmetric C-O-C glycosidic band stretching vibrations caused the peak at 1164 cm^−1^ [34], while the peaks seen at 1119 cm^−1^ and 1061 cm^−1^ were caused by the stretching of the C-O bond [35,36]. The recorded spectra of the investigated samples show certain structural variations in addition to their remarkable similarity. For instance, the FTIR spectra of the S and E samples show a second peak at about 1640 cm^−1^, which may be the result of the bending of adsorbed water, while the absence of this signal in the spectra registered for C samples and raw cotton indicate that the water was efficiently removed from these materials.

The lack of peaks at 1726 (C=O), 1550, and 1533 cm^−1^ (carboxylate doublet) [37,38] suggests that the samples are free of hemicellulose and lignin units, and the FTIR spectra support the purity of cellulose in all the analyzed samples.

### 4.3. Raman Spectroscopy

**Figure 4:**
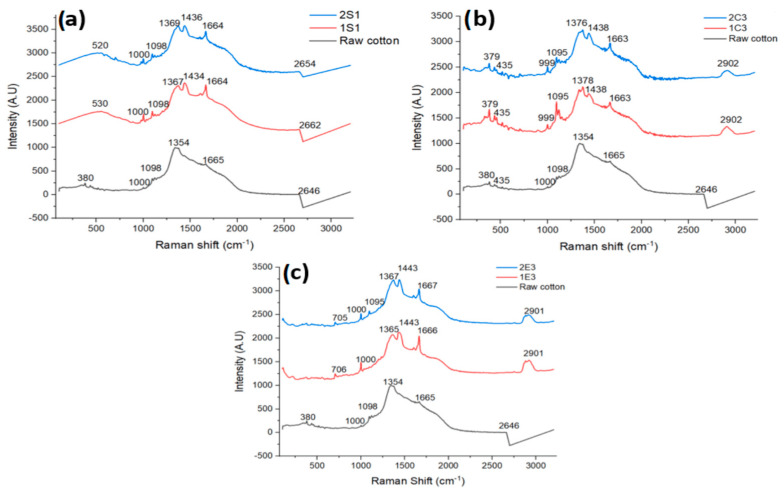
Raman spectra of CNC samples obtained through (**a**) H_2_SO_4_ hydrolysis; (**b**) HCl and (**c**) Enzymatic hydrolysis along with raw cotton.

Raman spectroscopy is a chemical analysis method that may be used to identify and measure the materials by the scattering of visible or near-infrared light from a laser source. Based on the molecular structure and forces, a sample’s vibrational spectrum may be assessed in Raman spectroscopy as a distinct “fingerprint”. Cellulose nanomaterials can be analyzed and characterized through Raman spectroscopy, which offers very important information about the crystallinity of the cellulose nanomaterials, and the detection of cellulose II or sulfate esters in CNC surfaces [39]. Figure 4a–c shows the Raman spectra of all the examined materials as well as raw cotton.

The Raman spectra of CNC samples obtained through H_2_SO_4_ hydrolysis are shown in Figure 4a. C-O-C glycoside linkage and C-C-C ring deformation bending are seen in samples 1S_1_ and 2S_1_ at 520 cm^−1^ and 530 cm^−1^, respectively [40], whereas the signal around 1000 cm^−1^ suggests stretching, C-C, and C-O: in-plane rocking (CH_2_) [41]. Cellulose ethers are identified by the intensities of peaks around 1100 cm^−1^ in the region of the C-C and C-O single bonds [42]. H-C-C, H-C-O, and H-O-C stretching and bending in-plane scissoring is indicated by vibrations between 1337–1378 cm^−1^ (CH_2_) [43]. CH deformation intensities are seen in samples 1S_1_ and 2S_1_ at 1434 cm^−1^ and 1436 cm^−1^, respectively [44]. The amide bond [45] corresponds to the signal at 1664 cm^−1^, which may be found in the amorphous region of CNC samples in the form of pectin residue. C-H and O-H stretches are seen in the 2500–3700 cm^−1^ area of the Raman spectra of CNC samples [46].

The Raman spectra of samples produced by HCl hydrolysis are shown in Figure 4b. Samples 1C_3_ and 2C_3_ showed vibrations at around 435 cm^−1^ and 379 cm^−1^, respectively, which were caused by the deformation of the C-C-C and C-C-O cellulosic rings [47]. The same peak intensities at 999 cm^−1^ in both samples suggest stretching, and C-C and C-O in plane rocking (CH_2_) [41]. Peak intensities at 1095 cm^−1^ can be linked to cellulose ether’s unique properties [42]. The signals from 1374–1380 cm^−1^ indicate stretching HCC, HCO, and HOC bending in-plane scissoring (CH_2_) [48]. CH deformation intensity is recorded at 1438 cm^−1^ for both samples [44]. The amide bond is indicated at 1663 cm^−1^ [45]. Both in Raman and FTIR spectra CH stretching (2800–3000 cm^−1^) intensities are observed around 2900 cm^−1^ [44].

The Raman spectra of samples produced by enzymatic hydrolysis are shown in Figure 4c. The C-S stretch of two methionine groups, which are present as enzyme residues in cellulose samples, is responsible for the weak band in the 1E_3_ and 2E_3_ samples at 700 cm^−1^ [49]. We also found vibrations in the E samples that were comparable to those in the S and C samples at roughly 1000 cm^−1^, indicating stretching between C-C and C-O: in a rocking plane (CH_2_) [41]. The characteristics of cellulose ethers, which are formed by the reaction of cotton with an alkaline solution such as those seen in S and C samples, can be attributed to the vibration at 1095 cm^−1^ [42]. We observed Raman shifts at about 1367 cm^−1^, which are caused by vibrations of CH_2_ [50]. CH deformation intensities are seen in both samples at 1443 cm^−1^ [41]. For samples 1E_3_ and 2E_3_, we noticed vibrations at 1666 cm^−1^ and 1667 cm^−1^, like in earlier samples, suggesting the presence of an amide bond in the amorphous portion of both samples. Vibration at 2901 cm^−1^ indicates CH stretching [44].

In addition to the existence of tiny quantities of lignin and ether, all spectra in Raman and FTIR confirm the characteristic bond in CNC samples and demonstrate the partial efficiency of CNC extraction from cotton.

### 4.4. XRD

**Figure 5:**
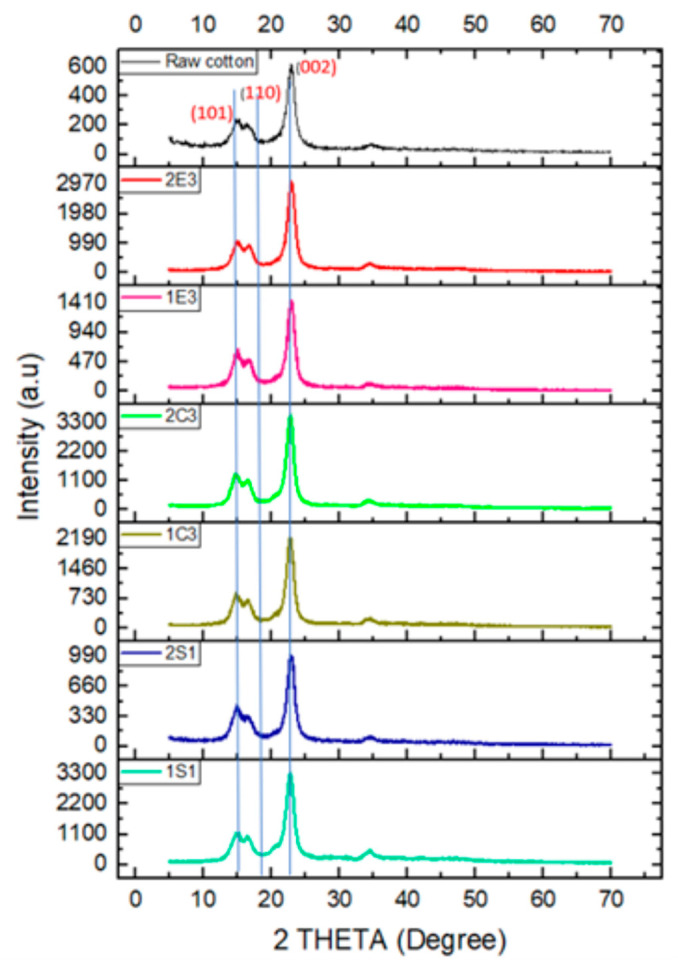
X-ray diffraction spectra of all CNC samples and raw cotton, respectively.

The X-ray diffraction patterns for raw cotton and samples that were prepared using various techniques are shown in Figure 5. Due to the phase transition of cellulose during the hydrolysis process, all samples exhibit peaks associated with crystalline cellulose, although the strength of these peaks varies between samples.

The primary crystalline plane (0 0 2) diffraction for nanocellulose samples was identified at 22.5° [51]. The intensity of the peaks for the (0 0 2) plane (2θ = 22.5°) for all CNC samples is higher than that of the raw cotton sample. This indicates that in all samples the crystallinity is higher than that of raw cotton regarding the (0 0 2) plane. The same phenomena are observed for the (1 0 1) plane (2θ = 14.7°) also. We have used two different methods to calculate the % of crystallinity of the samples.

(1) The integration method [52]: The integration method, which compares the area under the crystal peaks to the area under the whole curve using the following equation, can be utilized to determine the percentage of crystallinity of samples.
  (1) $$\%Crystallinity=\frac{area\;under\;the\;crystalline\;peaks}{area\;under\;all\;peaks}\times 100$$
(2) Segal’s method [51]: The intensities of the crystalline and amorphous regions can be used in Segal’s method, where I (Crystalline) is the maximum peak intensity of the crystallinity peak at 2θ around 22.5° or peak intensity at the plane (200), and I (amorphous) is the minimum peak intensity at 2θ around 18.2° or minimum peak intensity at the valley between planes (0 0 2) and (1 1 0) shown in Figure 5 and the values of calculated % crystallinity through the two methods are reported in Table 5.
  (2) $$\%Crystallinity=\frac{I\left(Crystalline\right)-I(Amorphous)}{I(Crystalline)}\times 100$$

In the H_2_SO_4_ hydrolysis method, the sample 1S_1_ has higher crystallinity than 2S_1_ (72.4% and 67.8%, respectively), indicating that the 1st bleaching method (20% (w) H_2_O_2_ and 4% (w) NaOH) has more effect on the crystallinity of the samples than the 2nd bleaching method (16% (w) NaOCl and 4% (w) NaOH) because all the other factors are the same for both samples (concentration of acids, reaction time, and temperature). A higher crystallinity value (77%) was reported for cellulose nanocrystals that were obtained from wood chips [53], but due to the use of various cellulosic materials, the reported Segal’s techniques was 90% in the same paper, which is also higher than our results (86% and 82%). Nevertheless, in the HCl hydrolysis procedure, sample 2C_3_ exhibits somewhat more crystallinity than the sample 1C_3_ (76.4% and 73.1%, respectively) based on the integration method which is relevant to Segal’s method (89.1 and 87.9, respectively) and also a similar result has been found by Yu et al. [51]. In the enzymatic hydrolysis method, sample 2E_3_ shows much more crystallinity than sample 1E_3_ (75.1% and 63.5%, respectively), but in Segal’s method, we have found around 84% crystallinity, which is very similar to the value that is reported by Camargo et al. [54] that had 83.7% crystallinity by using the enzymatic hydrolysis method from sugarcane bagasse. All these results show that the 2nd bleaching method is more effective than the 1st bleaching method in C and E samples in terms of crystallinity. It was found that the bleaching methods had an impact on the crystallinity of the samples.

### 4.5. Scanning Electron Microscopy (SEM)

**Figure 6:**
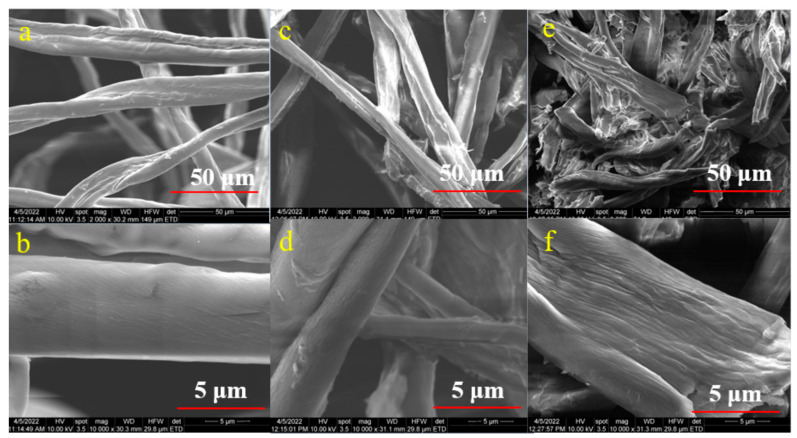
SEM Micrograph of the samples (**a**,**b**) raw cotton (**c**,**d**) 1E_3_, (**e**,**f**) 2E_3_.

**Figure 7:**
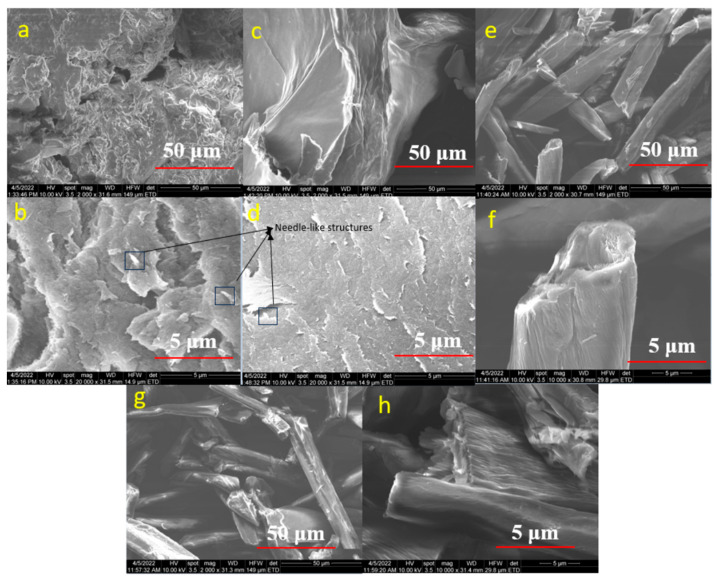
SEM Micrograph of the samples (**a**,**b**) 1S_1_, (**c**,**d**) 2S_1_, (**e**,**f**) 1C_3_, (**g**,**h**) 2C_3_.

Figure 6 and Figure 7 illustrate the morphology of the CNC samples made from raw cotton and the CNC samples obtained from acid and enzymatic hydrolysis. The morphology of raw cotton before bleaching is shown in Figure 6a,b, and the morphologies of the 1E_3_ and 2E_3_ samples, which were obtained by enzymatic hydrolysis, are shown in Figure 6c,d and Figure 6e,f, respectively. This shows that the size of the sample’s fibrillated structure was reduced and that enzymatic treatment broke down the long fibrillar structure into individual fibrils, though the fibrils are still long. Figure 6e,f show the 2E_3_ sample has a higher fractionation of cellulose fibers than the 1E_3_ sample (Figure 6c,d). Both XRD and SEM images of E samples show that the 2nd bleaching method (NaOCl) has a greater effect on crystallinity and morphology than the 1st bleaching method (H_2_O_2_).

We may conclude that the cellulases and xylanases did not totally break the fiber length into extremely short pieces throughout the enzymatic process, just changing the smooth surface of raw cotton into a little rough and coarse structure. E samples can be named CNF (cellulose nanofibrils) instead of CNC (cellulose nanocrystals). A similar result was also reported by Zhai, X. et al., 2021 [55].

Figure 7a–d display the SEM micrographs of the 1S_1_ and 2S_1_ samples, respectively; both bleaching processes (H_2_O_2_ and NaOCl) encourage the removal of cotton’s lignin and hemicellulose components, resulting in a surface that is very rough and full of wrinkles. In addition, needle-like structures can be seen on the surfaces of both samples.

Figure 7e–h illustrate the morphology of 1C_3_ and 2C_3_ samples obtained from hydrochloric acid hydrolysis, showing that the procedure did not totally shatter the structure of cellulose, but on the surface of cellulose fibrils (Figure 7f,h), we can see rod-like structures rather than needle-like and fibrils are still long like E samples. Similar SEM pictures demonstrating the impact of acid hydrolysis have been published in the scientific literature [14].

### 4.6. TGA Analysis

**Figure 8:**
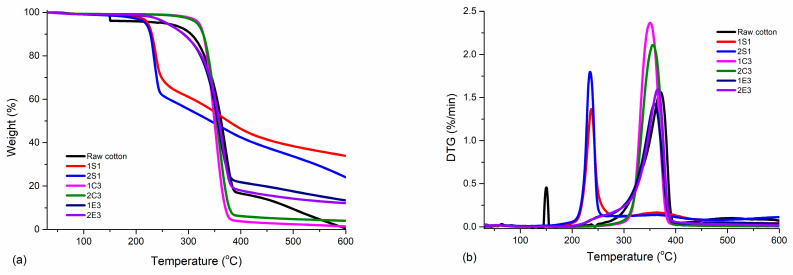
(**a**) TGA and (**b**) DTG profiles of the synthesized samples along with raw cotton.

The main thermal parameters from thermogravimetric investigations are reported in Table 6, while the TGA curve along with its derivative thermogravimetric curve (DTG) of each analyzed CNC product are reported in Figure 8a,b.

From the graphs, it is seen that all three types of samples (S, C, and E) have their own thermal characteristics. The first weight loss stage at 150 °C in the case of the raw cotton sample was most likely caused by absorbed water, followed by the start of degradation (10%) at 304 °C and the maximum degradation temperature at around 375 °C. The residue at 600 °C of the cotton was less than 0.5%, noting the complete degradation of the sample. For samples 1S_1_ and 2S_1_, the first mass loss of 2% is most likely caused by water and volatile compounds up to 100 °C, and the highest weight loss (up to 10%) is seen in the onset degradation temperature range of 214 °C to 230 °C for the sample 1S_1_ and 202 °C to 227 °C for the sample 2S_1_. The residue at 600 °C of the CNC was about 34% for 1S_1_ and 24% for 2S_1_ due to the inorganic sulfate groups and shows that the NaOCl bleaching sample would have lower residue than the H_2_O_2_ bleaching sample. Similar outcomes have been reported [56]; however, the presence of sulfate groups on the CNC surface is undesirable for particular applications since it accelerates decomposition at low temperatures [57].

For samples 1C_3_ and 2C_3,_ the maximum weight loss (up to 10%) is observed in the onset degradation temperature range from 300 °C to 326 °C for sample 1C_3_, and from 302 °C to 328 °C for sample 2C_3_. The residue at 600 °C of the CNC was about 1.4% for 1C_3_ and 4% for 2C_3_, revealing that there were no inorganic acids left behind after the combustion with oxygen gas. Similar work has been conducted [24] by using 12% HCl acid (~3.3 M) and 120 min hydrolysis time at 100 °C. T_max_ was found as 342 °C and the initial onset temperature was found at 292 °C, which corresponds to our results despite using different conditions.

For samples 1E_3_ and 2E_3_, the maximum weight loss (up to 10%) is observed in the onset degradation temperature range from 241 °C to 291 °C for sample 1E_3_, and from 241 °C to 290 °C for sample 2E_3_. The residue at 600 °C of the CNC was about 13% for 1E_3_ and 12% for 2E_3_, showing that there can be some buffer solution (sodium citrate) that had been used together with an enzymatic cocktail.

We may conclude that while the thermal stabilities of the 1S_1_, 2S_1_, 1E_3_, and 2E_3_ samples are comparable, the sulfate groups had a detrimental impact on the thermal stability of the CNC samples [58]. The slower rate of weight loss compared to the other samples in samples 1S_1_ and 2S_1_, however, may indicate that the sulfate groups in these samples may operate as flame retardants [59].

### 4.7. DSC Analysis

**Figure 9:**
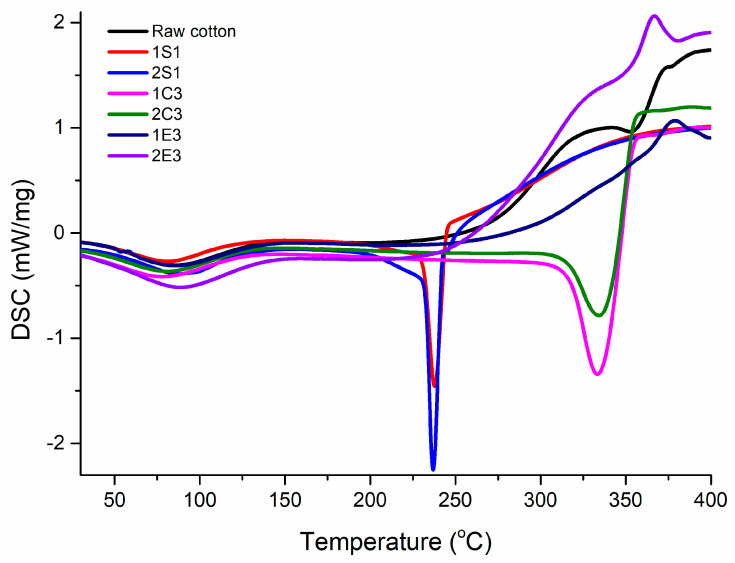
DSC profiles of all synthesized samples.

The thermal parameters calculated from DSC analysis of cellulose nanomaterials at a heating rate of 10 °C/min are presented in Table 7, and the obtained thermal curves are displayed in Figure 9.

Because surface water is becoming dehydrated, all thermograms display an endothermic event around 80 °C, which corresponds to the evaporation of absorbed water. This is typical behavior of cellulosic materials due to the fact that there are multiple interactions between the hydroxyl groups from its surface and water molecules [60].

The enthalpy for the melting transition and dehydration of the samples were calculated with the aid of the following equation.

(3) $$Heat\;of\;melting\left(\frac{J}{g}\right)=\frac{Area\;under\;the\;melting\;peak\left(J.\frac{{}^{\circ}C}{g}\right)}{Heating\;rate\left(\frac{{}^{\circ}C}{s}\right)}$$

According to the thermograms registered in Figure 9, it can be observed that the obtained S, C, and E samples exhibit their own thermal characteristics. Large endothermic peaks around 80 °C for all nanocellulose samples demonstrate the heat absorption phenomenon for the water evaporation process [61]. Sample 2S_1_ has a water-loss temperature of 86.3 °C and an enthalpy of dehydration of 52.1 J/g, showing that there is a stronger interaction of water than in sample 1S_1_, which has a water-loss temperature of 81.8 °C and an enthalpy of dehydration of 47.6 j/g. Similarly, the temperatures at which water is lost from samples 2C_3_ and 1C_3_ are 80.7 °C and 77.4 °C, respectively, and the enthalpy of dehydration are 61.7 j/g and 52.2 j/g, respectively. These results confirm that the NaOCl bleaching method for both acid hydrolysis presents higher water-loss temperature and enthalpy of dehydration than the H_2_O_2_ bleaching method. According to the melting point (T_m_) of the crystalline area of cellulose nanomaterials, an endothermic event is seen at around 237 °C for 1S_1_ and 2S_1_ samples and at about 333 °C for 1C_3_ and 2C_3_ samples. These observed differences in T_m_ in the case of C (T_m_ = 333 °C) and S samples (T_m_ = 237 °C) may be explained by the requirement of less activation energy to initiate the thermal decomposition of S sample because of the addition of sulfate groups [62].

In the case of 1E_3_ and 2E_3_ samples, an exotherm peak at 381 °C and 365 °C, respectively, is noted. This crystallization peak can be observed due to the rearrangement of molecules in a tighter network system, resulting in a certain amount of heat being released. Due to the significant number of amorphous areas in the materials or possible contaminants, the thermograms of these samples lack particular melting points and melting enthalpies. Both E samples showed approximately the same dehydration temperature but the enthalpy of dehydration of the sample 2E_3_ is much higher than that of the sample 1E_3_ (80 °C and 65.1 °C, respectively) indicating a higher water content than its counterpart. These results implied that different acid and enzymatic hydrolysis methods along with bleaching techniques could greatly affect the thermal degradation of nanocellulose.

## 5. Conclusions

Cellulose nanomaterials (CNC and CNF) were successfully extracted from raw cotton via hydrogen peroxide and sodium hypochlorite bleaching method followed by acid and enzymatic hydrolysis. Thermogravimetric and morphological parameters, crystallinity, zeta potential, and hydrodynamic properties of the materials were examined. The separation of straight fiber bundles into coarse and shorter CNC particles was seen in an SEM micrograph, and numerous wrinkles and needle-like structures were seen on the surface of S samples. FT-IR and Raman spectra of CNCs and CNFs displayed partial removal of lignin and hemicellulose domain from raw cotton. The Zeta potential value of the 1S_1_ sample is more negative than the 2S_1_ sample (−42 mV and −32.8 mV, respectively), showing that the H_2_O_2_ bleaching method is more effective than the NaOCl method. On the other hand, the 1S_1_ and 2S_1_ samples have the smallest particles length, 97.91 and 77.17 nm. By using the integration technique, XRD revealed that the percentage of crystallinity of the samples ranged between 67.8% and 76.4%; however, using Segal’s method, the percentage of crystallinity of the samples ranged from 82 to 89%, depending on the type of hydrolysis and bleaching procedure. The TGA analyses showed that C and E samples presented higher thermal stabilities than S samples, while the bleaching method did not significantly affect the thermal properties of samples but impacted the hydrodynamic characteristics and crystallinity of CNC samples. The second bleaching method (NaOCl) has a stronger influence on crystallinity and morphology than the first bleaching method (H_2_O_2_), according to XRD and SEM pictures of E samples. SEM analysis demonstrated a partially efficient production of CNC from cotton by sulfuric acid hydrolysis, as well as CNF formation through hydrochloric acid and enzymatic hydrolysis.

## Figures and Tables

**Table 1:** Hydrolysis of cotton samples with sulfuric acid in different concentrations and times.

Bleaching	wt.% of H_2_SO_4_	Hydrolysis Time (Minute)	Temperature (°C)	Abbreviation of Sample
1st method: 20 wt.% H_2_O_2_ and 4 wt.% NaOH	60	10	70	1S_1_
	50	20	70	1S_2_
	40	30	70	1S_3_
2nd method: 16 wt.% NaOCl and 4 wt.% NaOH	60	10	70	2S_1_
	50	20	70	2S_2_
	40	30	70	2S_3_

**Table 2:** Hydrolysis of cotton samples with hydrochloric acid at different concentrations and times.

Bleaching	wt.% of HCl (M)	Hydrolysis Time (Minute)	Temperature (°C)	Abbreviation of Sample
1st method: 20 wt.% H_2_O_2_ and 4 wt.% NaOH	20	30	75	1C_1_
	15	60	75	1C_2_
	10	90	75	1C_3_
2nd method: 16 wt.% NaOCl and 4 wt.% NaOH	20	30	75	2C_1_
	15	60	75	2C_2_
	10	90	75	2C_3_

**Table 3:** Hydrolysis of cotton samples by using enzymes in different concentrations and times.

Bleaching	Xylanase (290,000 U/g)	Cellulase (500,000 U/g)	Hydrolysis Time (h)	Temperature (°C)	Abbreviation of Sample
1st method: 20 wt.% H_2_O_2_ and 4 wt.% NaOH	40 mg	160 mg	24	50	1E_1_
	30 mg	120 mg	24	50	1E_2_
	20 mg	80 mg	24	50	1E_3_
2nd method: 16 wt.% NaOCl and 4 wt.% NaOH	40 mg	160 mg	24	50	2E_1_
	30 mg	120 mg	24	50	2E_2_
	20 mg	80 mg	24	50	2E_3_

**Table 4:** The stability of colloids is based on the zeta potential value [29].

Zeta Potential (mV)	Stability Behavior
0 to ±5	Rapid coagulation
±10 to ±30	Incipient instability
±30 to ±40	Moderate stability
±40 to ±60	Good stability
>61	Excellent stability

**Table 5:** % crystallinity of samples by using integration and Segal’s method.

% Crystallinity		
Samples	Integration Method	Segal’s Method
1S_1_	72.4	86.2
2S_1_	67.8	82.0
1C_3_	73.1	87.9
2C_3_	76.4	89.1
1E_3_	63.4	84.9
2E_3_	75.1	84.6
Raw cotton	40.6	81.0

**Table 6:** Percentage decomposition of samples at different temperatures.

Samples	T_d3%_ (°C)	T_d5%_ (°C)	T_d10%_ (°C)	T_max_ (°C) from DTG	Mass Loss (%)
Raw cotton	150	255	305	375	99.57
1S_1_	214	222	230	236	66.16
2S_1_	202	218	227	235	76.17
1C_3_	309	320	329	350	98.62
2C_3_	302	317	328	355	96.02
1E_3_	241	259	291	358	86.69
2E_3_	241	259	290	364	87.93

**Table 7:** The most important thermal parameters extracted from DSC analysis of all samples.

Samples	T_d_ (°C) ^a^	ΔH_Td_ (J/g) ^b^	T_m_ (°C) ^c^	ΔH_m_ (J/g) ^d^
Raw cotton	86.1	74.9	352.3	37.4
1S_1_	81.8	47.6	237.6	70.4
2S_1_	86.3	52.1	236.8	72.1
1C_3_	77.4	52.2	333.4	232.9
2C_3_	80.7	61.7	334.3	208.1
1E_3_	86.2	65.1	-	-
2E_3_	86.1	80.0	-	-

(a—dehydration temperature; b—enthalpy of dehydration; c—melting point of the crystalline region of CNC; d—enthalpy of melting).

## Data Availability

The raw data required to reproduce these findings can be shared at request.
